# Exergaming for Children and Adolescents: Strengths, Weaknesses, Opportunities and Threats

**DOI:** 10.3390/jcm7110422

**Published:** 2018-11-08

**Authors:** Valentin Benzing, Mirko Schmidt

**Affiliations:** Institute of Sport Science, University of Bern, 3012 Bern, Switzerland; mirko.schmidt@ispw.unibe.ch

**Keywords:** active video gaming, serious games, physical activity, physical exercise, sedentary behavior, narrative review

## Abstract

Exergaming, or active video gaming, has become an emerging trend in fitness, education and health sectors. It is defined as digital games that require bodily movements to play, stimulating an active gaming experience to function as a form of physical activity (PA). Since exergaming is becoming more popular, claims have been made on the usefulness of exergaming. It has, for example, been entitled as being “the future of fitness” by the American College of Sports Medicine, promoting PA and health in children and adolescents. However, research also suggests that long-term engagement in exergaming is difficult to achieve, and there is a noticeable reservation towards exergaming by parents, teachers and caregivers. To provide an overview and to outline the future directions of exergaming, the aim of this review was to critically illustrate the strengths, weaknesses, opportunities and threats of exergaming to promote PA and health in children and youth. The available evidence indicates that exergaming has the potential to improve health via an increase in PA. However, it seems that this potential is frequently underexploited, and further developments such as customized exergames are needed.

## 1. Introduction

Across the globe, the majority of adolescents are not reaching the recommended amount of physical activity [1], consequently impacting their physical and mental health [2,3,4]. Reasons for decreased physical activity levels may be caused by various factors, including the fact that children and adolescents spend much more time sedentary in front of the screen than in the past [5]. Because of the increased sedentary screen time, exergaming (or active video gaming) might bear potential for making children and adolescents more active, and thus positively affecting their health [6]. Therefore, the question is whether exergaming can indeed positively affect sedentary behavior and health in children and adolescents. Before discussing the effects of exergaming in children and adolescents on physical activity and health, several general issues must be addressed.

Firstly, it is important to outline what comes under the umbrella term of exergaming. To date, there is no universal definition of exergaming available. According to Bogost [7] exergaming has been labeled by the media as “the combination of exercise and videogames”. Although this description has been used by both the commercial industry and in the scientific field [8,9], it does not serve as a suitable formal definition. This becomes apparent when adhering to the traditional definition of “exercise”, as intentionally to improve or maintain physical fitness with a planned, repetitive, and structured format (Caspersen et al. [10]), according to which many available exergames (for example, those with alternative intentions than improving fitness) would be excluded [11]. To overcome this issue, broader definitions have been used, describing exergaming as “interactive video gaming that stimulates an active, whole-body gaming experience” [6], or according to Gao et al. [12], it “refer[s] to videogames that require bodily movement to play and function as a form of physical activity”. Since some exergames do not necessarily involve whole-body movements, and considering that there is potential for the focus to be not only on the visual but on the tactile or auditory domains in the future, we propose to combine both definitions, and exchange videogames with digital games. Consequently, for the purpose of the current analysis, the term exergames will refer to digital games that require bodily movements to play, stimulating an active gaming experience to function as a form of physical activity.

Although exergames have been available since the 1980s [6], in research it has received increased attention only in the last ten years [13]. Since then, the number of articles published related to exergaming or active video gaming has substantially increased. Although exergaming is still in its infancy, several reviews and meta-analyses regarding its potential usefulness to promote physical activity and health have already been published [14,15]. The majority of these studies have been performed within the health sector, in particular public health and rehabilitation [16]. Besides medicine, however, other disciplines such as psychology, sport science, neuroscience or computer science are also involving exergames in their research, showing the interdisciplinary nature of this intervention.

Exergaming is diverse. Reflecting the manifold research disciplines, exergaming is applied in many fields of application, including prevention [17], treatment [18] and rehabilitation [19,20]. Application also reaches a vast range of individuals, including both clinical and non-clinical populations [21]. This diversity is also exhibited in the target age groups, ranging from young children [6] to the elderly [22]. Since children and adolescents seem particularly attracted to video games, these age groups have been identified as a group who will have “special interests in and benefit from exergames” [23]. Consequently, besides the elderly, exergaming research has primarily focused on younger age groups.

**Figure 1:**
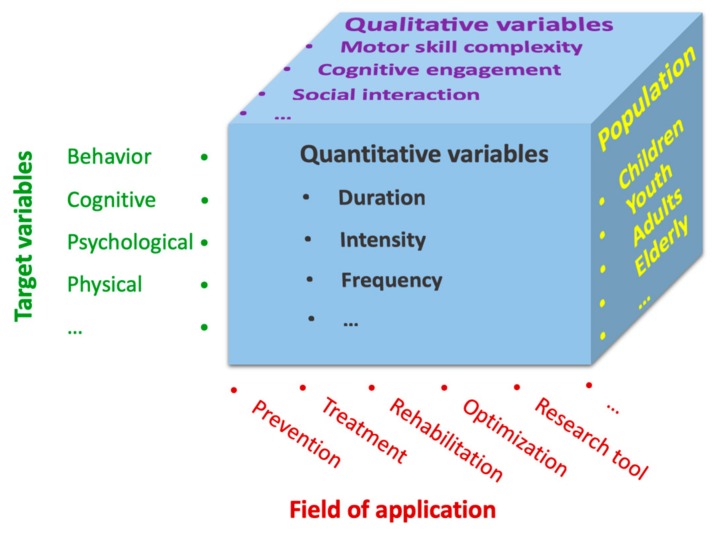
Overview of different dimensions associated with exergaming.

Moreover, similarly to traditional exercise [24,25], exergames vary in quantitative and qualitative exercise characteristics. According to Pesce [25], quantitative parameters such as duration and intensity focus on a “medical” perspective, examining dose-response relationships. Whereas, qualitative parameters, which are globally defined as type or mode of exercise, reflect non-physical aspects of exercise tasks, such as cognitive or coordinative demands [25]. In exergaming an interplay of these factors (see Figure 1) may be crucial with regard to eliciting benefits in physical, cognitive and psychosocial target variables. In summary, exergaming may be considered as multifaceted and dependent on a variety of factors.

The dramatic increase in interest associated with exergaming has risen due to a variety of factors, including the technical advances which have enabled individuals to play exergames at home, and because it bears unique strengths and opportunities, such as specificity and adaptivity. In contrast to these positive factors, however, there is an uncertainty (e.g., from parents, teachers etc.) regarding the usefulness of exergaming when considering the specific weaknesses and threats associated with use in pediatric populations. Therefore, the aim of this review was to give an overview on the Strengths, Weaknesses, Opportunities and Threats (SWOT) associated with pediatric exergaming. Since exergaming is a highly diverse and a rich topic (see Figure 1), we have limited this review to include the most important dimensions for children and adolescents, basing our work on examples relevant to this population and their physical activity and health.

## 2. Strengths

**Figure 2:**
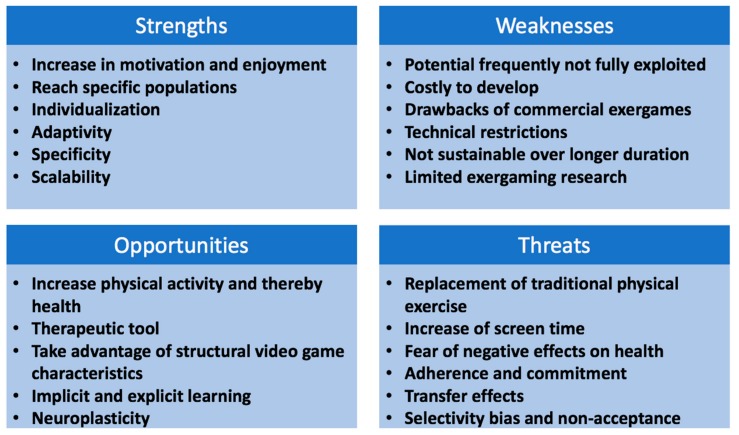
Strengths, weaknesses, opportunities and threats associated with exergaming in children and adolescents.

One of the greatest strengths of exergaming (for an overview about strengths, weaknesses, opportunities and threats see Figure 2) is that it seems to increase the motivation and engagement in physical activity [26,27]. This is supported by results from previous research, demonstrating that exergaming elicits motivational gains, as well as flow, immersion and enjoyment [28,29]. The importance of enjoyment is gaining more attention in exergaming research because it seems to be an important variable in sustaining a higher physical activity level [23]. Moreover, a greater enjoyment within physical activity has been found to be important for cognitive benefits, which in turn are thought to positively influence academic achievement [30]. Although, it is currently unclear which game design characteristics maximize motivation and enjoyment, it is assumed to be important in explaining why it is easier to achieve a certain physical activity level when using the right exergame [31].

Another advantage of exergaming is that it helps to reach specific populations. This might be particularly important for children who are not meeting the recommended amount of physical activity through traditional methods, or who spend much time playing video games. In this way, it has been shown that exergaming bears the potential to reduce obesity [13,17]. Exergaming can be integrated into the school curriculum, contributing to getting children active, and consequently promoting positive effects on body mass index and fitness [26,32,33]. Moreover, children with attention deficit hyperactivity disorder, for example, who spend more time playing sedentary video games than typically developing children, might find exergames to be a viable option to replace sedentary screen time [34]. Therefore, due to the attraction of video games in specific populations, it is possible that children who are more sedentary could be reached to increase physical activity, resulting in a variety of physical and cognitive health benefits [35].

Another strength of exergaming is that it allows for individualization and adaptivity [36]. In this way, an exergaming session may be tailored to fit the needs of an individual. So, for example, important characteristics of the child such as the fitness level can be taken into account to avoid under- and overload [37]. Moreover, not only an initial assessment, but continuous measurement and adjustment can be completed with exergaming. An adjustment during the training by an algorithm combined with immediate feedback to the individual has the potential to ensure that the individual always trains at the “sweet spot”. So, for example, physical and cognitive challenge may be monitored and adjusted, assuming that well-adjusted physically and cognitively engaging physical activities provide more training gains [9]. Thus, not only physical but also mental benefits may be enhanced, which in turn holds relevance for promoting academic achievement [25,38,39], creating an additional benefit of exergaming [40].

Specificity is a further advantage of exergaming. In a virtual environment, there are unlimited opportunities to create exergames for specific training purposes e.g., isolated movements may be trained and repeated in an infinite number of trials. Using such adaptations ensures that skill development in physical education [37] or in elite sports [41] can be trained. However, currently the most frequent implementation of specificity is in rehabilitation. The benefits of specificity in this field is demonstrated by studies which show that exergaming improves gross motor skills of atypically developing children [42], acting as a useful rehabilitation tool in this population (e.g., balance control) [43]. Moreover, task-specific or outcome-specific trainings seem to be a prerequisite for positive effects [44,45].

Another strength is the high scalability and economic feasibility of exergames. Most exergames use commercial game consoles and can be connected to a conventional TV screen. Therefore, exergaming can be applied almost anywhere, at any time. Since many families in developed countries have their own TV at home, and some even a game console, this results in high scalability, and the potential for distribution to reach many households.

In summary, exergaming has the potential to increase physical activity, and thus positively impact physical, cognitive and psychosocial target variables [46]. Moreover, certain populations are particularly likely to benefit from exergaming, for example, children who are inactive and not interested in traditional exercise [23]. Thus, it can be concluded that with exergaming, increased media consumption doesn’t necessarily have to mean reduced physical activity. Having this in mind, exergaming could provide a useful adjunct to traditional methods of physical activity, however, there are also several potential weaknesses associated with exergaming that should be taken into consideration.

## 3. Weaknesses

It could be argued that one of the greatest weaknesses of exergaming is that its potential is frequently underexploited. When considering the aforementioned strengths, it becomes clear that to obtain the most beneficial effects, exergames have to be tailored to the target population, as well as the target variables [36]. However, tailoring exergames is costly and takes a long time to develop, therefore this is not often done. Although alternative factors (such as behavior change procedures) that enhance the effects of exergames and may also be applied to commercially available games have been proposed [47], more systematic collaborations between science and industry are needed.

The technical capabilities and resources of professional game publishers are substantial, consequently leading to more sophisticated, entertaining and appealing exergames. These more sophisticated exergames might be advantageous with regard to fun and enjoyment, however, there are also drawbacks specific to commercial exergames. Besides less individuality, adaptivity and specificity of these untailored exergames, generally it is difficult to access the data of the exergame, for example, to control for fidelity of implementation and energy expenditure consumption [48]. This makes it difficult to fully exploit the benefits of exergaming as a monitoring tool, for example in clinical populations or in a school setting.

Another technical consideration is that the gaming experience as well as the elicited energy expenditure of exergames is highly dependent on the virtual environment as well as on the technical capabilities, such as the sensors [47,49]. This is reflected in the finding that exergames involving lower body movements elicit more energy expenditure, thus both the virtual environment and the technical implementation is important [49]. Although there are several different sensor technologies available, including hand-held motion sensors as well as motion capture technology, there are problems attached to both. Hand-held motion sensors make it easy to cheat, whereby the computer believes that the child is engaging in the physical activity when they are in fact stationary [47]. For motion capture technology, although it seems to encourage more energy expenditure [50], it was found to be error-prone [51]. This in turn is problematic with regard to adherence and drop-out, where technical difficulties discourage the user from participation [51,52].

Besides technical issues, it seems that current exergames are not able to keep the interest of players over longer time periods [53]. This can be explained by the reasons frequently given for dropout in exergaming interventions, indicating that the played exergames became boring [51,52]. This points to a lack in variety and immersion in the currently available exergames. Although dropout rates have been found to be smaller in multiplayer modes, it seems that current exergames are not sustainable in general. Thus, although exergaming can elicit moderate to vigorous intensities [54], other factors increasing adherence have to be considered, and sustainable play is yet to be proven [55].

Considering that exergaming has only been developed in the last decades, there has already been a great amount of research done so far. Nevertheless, exergaming research is limited, and there are many issues that remain unclear. For example, it is unknown whether exergaming will replace traditional exercises or sedentary behaviors (see threats section). This is of great importance considering that a frequent goal of exergaming is to increase physical activity levels. Moreover, exergaming has rarely been compared or combined with traditional exercises in children [33,56], and it is unclear whether exergaming results in as much physical activity as traditional exercises, and also how long potential positive effects of exergaming persist [57,58,59]. Since exergaming is highly diverse, and most studies apply different games, more studies investigating the underlying mechanisms as well as the specific exergaming characteristics and their impact on physical activity levels are needed.

Taken together, the potential of exergaming is frequently underexploited, and in order to make the best use of exergaming in the future, more games should be customized to meet the needs of specific populations [36]. Although customizing exergames is currently costly and limited by technical considerations, exergaming technology is advancing and becoming more affordable, creating potential opportunities for future development.

## 4. Opportunities

Although in exergaming research, a variety of strengths have already shown its potential, a major opportunity of exergaming is to further develop these strengths. So, for example, a major opportunity of exergaming (which has been shown to some extent) is that it helps to get children and adolescents active, which would promote motor skills, cognitive performance as well as mental health. Physical inactivity is a major health factor in children and adults due to the many harmful effects on both physical [3] and mental health [4]. Considering that most children and adolescents in Europe and the US play video games [60], exergaming could become an important tool to promote physical activity, reach individuals that could not be reached by alternative methods, and promote positive effects on cognitive performance, mental and physical health [46].

The use of video games as a therapeutic tool is promising [61]. Children and adolescents in clinical as well as rehabilitative settings may benefit from exergaming. It has already been shown in rehabilitation that exergaming has the potential to improve general as well as disease specific outcomes [44]. Moreover, there is promising evidence showing that exergaming is feasible as a physiotherapeutic intervention in adults [62], and that children’s motor learning may even be enhanced by practice in a virtual environment [63]. Thus, in the future, exergaming may be used by occupational or physiotherapists to reach those who cannot do traditional physical therapy due to their physical condition or location. Since exergaming is highly scalable, it could be utilizable for a variety of diseases and disorders in the future. In this way, exergaming could be used as a form of therapy/rehabilitation to help to get many children active who cannot be reached, due to their distant location for example, or their inability to leave the hospital due to their disease [64].

Additionally, the applied exergames could serve as a continuous diagnostic tool. While playing, information about the state of the user (including psychophysiological data) could be logged. The computer or a caregiver could then access the diagnostic information and automatically or manually adjust the exergame to suit the needs of the individual [36]. Furthermore, it is probable that in the future, multiple technical devices access and share their data to provide an optimized training including exergaming, augmented reality and traditional exercises.

Several factors are suggested to be important for successful interventions. These factors include both hardware and software as well as many structural characteristics, which lead to both immersion and increased motivation to play video games [65,66]. In children and adolescents, a goal would be to use comparable output hardware as well as game elements [66] and structural characteristics of video games [65] in exergaming. In further detail, these characteristics could include multiple levels, such as social, manipulation and control, narrative and identity, reward and punishment and presentation features [65]. A systematic inclusion of these characteristics could help to enhance exergaming, facilitate initiation and maintenance and finally replace sedentary behavior with physical activity.

As another opportunity, learning (of motor skills for example) can occur both implicitly and explicitly in exergaming, since it is possible to learn movements embedded in a story without formal instruction [45]. This combination of playful non-instructive learning with informative instructive learning bears great potential for children and adolescents, but must be investigated further in the future.

Moreover, exergaming has the potential to stimulate neuroplasticity through environmental enrichment and task complexity. Neuroplasticity refers to “the capacity of the nervous system to modify its organization” [67]. These adaptations for example can occur as a consequence of skill training, resulting in learning and skill acquisition [68]. Since research has shown that environmental enrichment seems to stimulate cognitive functions [69] as well as neural and synaptic growth [70], exergaming consequently has the potential to promote neuroplasticity. However, not only novel or enriched environments, but also physical exercise [68] and task complexity [71] seems to facilitate neuroplasticity. Since as mentioned before, a strength of exergaming is adaptivity, task complexity can be kept on a (optimal) challenging level in a changing virtual environment, altogether contributing to facilitated neuroplasticity.

Considering the mentioned opportunities, it is suggested to specifically implement existing strengths of exergames such as adaptivity, specificity and individuality in a more systematic fashion. Furthermore, to enhance potential benefits and increase adherence, one could include structural video game characteristics as well as sound exergaming procedures, such as involving parents in game play [47,65,72]. However, these advances might also be associated with a variety of new and unexpected threats which should be considered.

## 5. Threats

The most likely risk of exergaming, whilst also being responsible for some of the greatest strengths, is that exergaming takes place in a virtual environment. Because of this circumstance, there are concerns that exergaming replaces traditional physical exercises and increases screen time.

One of the greatest threats of exergaming revolves around the question of whether traditional physical activity is replaced by exergaming. Given that in exergaming research a major aim is to increase physical activity levels and impact physical health (e.g., in obesity), a replacement of traditional exercises would have an adverse effect. There is first evidence in overweight and obese adolescent girls, indicating that an exergaming intervention may result in increased self-reported physical activity and less TV watching. However, the results are somewhat inconclusive since there was no increase in physical activity provided by accelerometry [73]. Nevertheless, in the scientific community, it is well established that exergaming should not, and cannot replace traditional physical exercise [46]. On the contrary, the underlying idea is that it should replace sedentary behavior such as video gaming. Since there is currently not enough scientific evidence on this issue, this risk must be researched in future studies and monitored carefully.

Whether exergaming increases screen time substantially is also unclear from a scientific standpoint. On the one hand, it is possible that applied exergaming interventions, in physical education (PE) for example, increase screen time to a smaller extent. On the other hand, research has shown that PE combined with exergaming revealed greater improvements with regard to fitness and BMI than PE alone [33]. Therefore, exergaming might be a gateway to sedentary people. Consequently, to fully evaluate the effects caused by the added active screen time, further research is needed to investigate both the positive effects of more physical activity and the negative effects evolving from more screen time.

Related to this issue, the underlying assumption of people who fear increases in screen time is that exergaming has negative effects on the mental health of children and adolescents. Here it is thought that exergames will cause social isolation due to excessive video gaming, or increase aggression [74]. This fear, however, is likely based on previous research about sedentary violent video games [75] and problematic gaming behavior [76]. In contrast to these findings, research also revealed positive effects of sedentary video games on cognition, motivation, emotion and social benefits [77,78], meaning that the hypothesized negative effects of sedentary video gaming are currently subject to controversial debate [79]. Most importantly, it is questionable whether the negative effects of specific types of sedentary video games can be easily transferred to active video games, since these games can only be played limitedly due to the level of physical exertion and reaching fatigue. Nevertheless, this fear leads to a reservation towards exergaming, which consequently threatens adherence and commitment.

The transferability from exergaming to the real world is uncertain. Another risk refers to the controversial debate in the cognitive training literature regarding the transferability of skills from a (virtual) training to the real world [80,81]. Since near transfer effects are easier obtained, the virtual environment can be systematically designed to come as close to real world settings as possible [41,82]. Although exergaming offers this unique potential to increase transfer effects compared with previous video games, it is also likely that transfer effects are limited by the choice of exergames. Thus, theory driven approaches [72] for example using the identical elements theory [83] in order to increase transfer effects are needed.

Finally, exergaming might attract certain individuals, which potentially introduces selectivity bias. Research has shown a non-acceptance of potential users because of the digital nature of the games [51]. Thus, due to the technical implementation of exergaming utilizing game consoles and the virtual environment, exergaming itself might lead to an attraction of certain individuals [34]. Since, for example, more boys are playing video games than girls [76], a risk might be that boys in particular are attracted by exergaming. This in turn could lead to a selectivity bias in research and practice [34]. Notably, as with every novel technology, exergaming must first prove its value to convince different potential users, parents, teachers and caregivers. Nevertheless, researchers and game developers should pay attention to the inclusion of different target groups.

Taken together, these potential threats indicate that exergaming should not be a substitute for traditional sports or exercises [46], and that an increase in screen time has to be monitored carefully in order to prevent potential negative effects. Among others, the most important future challenges include the increase of transfer effects by using theory driven approaches, such as the identical elements theory, which might help to specifically target intended outcome variables during exergaming [83].

## 6. Summary

The current SWOT analysis reveals that exergaming has a variety of strengths, most notably identifying the potential for individualization, adaptivity and specificity. Additionally, the increased motivation and engagement, and the potential to reach specific populations lay the foundations for exergaming to promote physical activity and health in children and adolescents successfully.

However, there are also weaknesses associated with exergaming, which mostly involve its implementation. In further detail, it seems that the potential of exergaming is frequently underexploited, and that in previous exergaming studies, there is limited systematic inclusion of theories and underlying mechanisms.

Since exergaming is a relatively new technological achievement, promising opportunities such as getting people active as well as promoting motor skills, cognitive performance and mental health warrant further investigation. However, substantial modifications must be monitored continuously and carefully regarding potential threats, most importantly considering the risk of replacement of traditional physical exercise, and the risk of increased screen time.

Taken together, since most children and adolescents in Europe and the US play video games [60], we are already past the point of no return. Thus, the question is no longer whether children and adolescents are playing video games and how can we prevent them from doing so, but how we can positively impact what type of digital games they use, and for what purpose they are playing. Considering this, exergaming could be a viable tool to positively influence the screen time experience of children and adolescents.

## 7. Limitations

This SWOT analysis suffers from weaknesses which are worth noting. First, it might be that strengths and positive effects are more likely to become published than null or even negative effects. Having this publication bias in mind, the current analysis might also lean towards the strengths of exergaming. Thus, more empirical research, not only investigating whether there is a positive effect or no effect of exergaming on specific variables, but specifically focusing on the potential negative effects is needed. Second, it is impossible to make a truly objective SWOT analysis [84]. One could even say that a SWOT analysis is subjective by definition, since a weighting on the importance of aspects must be done. Therefore, this analysis also contains subjective opinions. Third, the current SWOT analysis has no claim to be complete. The aim was to collect and identify the most important aspects, however, this review is not exhaustive.
